# Object representations in ventral and dorsal visual streams: fMRI repetition effects depend on attention and part–whole configuration

**DOI:** 10.1016/j.neuroimage.2011.04.035

**Published:** 2011-07-15

**Authors:** Volker Thoma, Richard N. Henson

**Affiliations:** a School of Psychology, University of East London, UK; b MRC Cognition and Sciences Unit, Cambridge, UK

**Keywords:** Attention, fMRI, Object recognition, Repetition suppression, Repetition enhancement, View-dependence

## Abstract

The effects of attention and object configuration on the neural responses to short-lag visual image repetition were investigated with fMRI. Attention to one of two object images in a prime display was cued spatially. The images were either intact or split vertically; a manipulation that negates the influence of view-based representations. A subsequent single intact probe image was named covertly. Behavioural priming observed as faster button presses was found for attended primes in both intact and split configurations, but only for uncued primes in the intact configuration. In a voxel-wise analysis, fMRI repetition suppression (RS) was observed in a left mid-fusiform region for attended primes, both intact and split, whilst a right intraparietal region showed repetition enhancement (RE) for intact primes, regardless of attention. In a factorial analysis across regions of interest (ROIs) defined from independent localiser contrasts, RS for attended objects in the ventral stream was significantly left-lateralised, whilst repetition effects in ventral and dorsal ROIs correlated with the amount of priming in specific conditions. These fMRI results extend hybrid theories of object recognition, implicating left ventral stream regions in analytic processing (requiring attention), consistent with prior hypotheses about hemispheric specialisation, and implicating dorsal stream regions in holistic processing (independent of attention).

## Research highlights

► FMRI repetition effects for visual objects were studied by varying view and attention. ► Attended objects in intact and split views showed fMRI suppression in ventral areas. ► View-specific fMRI enhancement was found in dorsal areas regardless of attention. ► Left ventral effects correlated with attended, dorsal effects with unattended priming. ► Left ventral areas are implicated in analytic, dorsal areas in holistic processing.

## Introduction

How do we recognise familiar objects when they are shown from an unfamiliar viewpoint, or with some of their parts obscured? In order to infer the nature of representations that mediate such object constancy, many behavioural studies have measured ‘priming’: the improved recognition performance associated with repetition of an object, as a function of various changes in the manner in which that object is depicted (Bartram, 1976). A general finding is that priming is greatest if an object is repeated in the same view, and decreases when shown in a different view, such as when rotated in the picture plane (Lawson, 1999; Thoma and Davidoff, 2007). Studies using functional magnetic resonance imaging (fMRI) also show repetition effects: Blood oxygen level-dependent (BOLD) signals in various ventral visual stream regions, such as in lateral occipital and inferior temporal cortices, tend to decrease when a visual object is repeated (Grill-Spector et al., 1999; Kourtzi and Kanwisher, 2000; Koutstaal, 2001; James et al., 2002; Vuilleumier et al., 2002). A number of fMRI experiments have shown that this “repetition suppression” (RS; Grill-Spector et al., 2006) is maximal when the initial and repeated views of an object are identical, and decreases with the amount of change in view, e.g. following rotation (Gauthier et al., 2002; Ewbank et al., 2005; Andresen et al., 2009). This finding has been used to support theories that objects are stored in “view-specific” representations, e.g., via several 2D views of an object (Ullman, 1989, 1998; Poggio and Edelman, 1990; Bulthoff and Edelman, 1992; Basri and Ullman, 1993; Tarr, 1995; Tarr and Gauthier, 1998). Object invariance is then accomplished by interpolation across these views (Ullman 1989; Poggio and Edelman, 1990; Logothetis, 1994), or by a distributed neural representation across view-tuned neurons (Perrett et al., 1998; for review, see Peissig and Tarr, 2007).

However, the simple rotation of object images in-plane or even in-depth tends to maintain some low-level similarity between initial and repeated images (e.g., even at the level of pixel overlap; Chouinard et al., 2008). A recent study by Hayworth and Biederman (2006) controlled for this by using part-based line-drawings of objects, the contours of which did not overlap with previously shown contours of each part. According to this study, more anterior parts of the ventral visual stream (specifically the posterior fusiform gyrus) are involved in an intermediate representation of shape that is largely “part-based”, and abstracted from view-specific, retinotopically-based representations. Part-based representations are often conceptualised as structural descriptions (Sutherland, 1968; Marr and Nishihara, 1978; Biederman, 1987), in which an object is stored as a combination of generalised parts and their spatial relations. Structural descriptions are largely viewpoint-independent as long as crucial parts are visible (Hummel and Biederman, 1992). There is considerable evidence for structural descriptions from behavioural and animal studies (Biederman, 1987; Biederman and Gerhardstein, 1993; Biederman and Bar, 1999; Lazareva et al., 2008). Importantly, structural descriptions also allow for effects of changed viewpoints on recognition of repeated objects, such as when in-plane rotations perturb the spatial relations between parts (Hummel and Biederman, 1992) or when in-depth rotations occlude or reveal parts across rotations (Biederman and Gerhardstein, 1993).

A recent model of object recognition, termed the “hybrid model” (Hummel and Stankiewicz, 1996; Hummel, 2001), proposes that objects are processed in two parallel routes: an “analytic” route, which uses view-independent structural representations in terms of an object's parts and their spatial relations, and a “holistic” route, which uses exclusively view-dependent representations with fast access to stored views. In the hybrid model, visual attention is necessary to bind parts and spatial relations within the analytic route, but is not required for processing in the holistic route (see Hummel, 2001, for details). Contrary to view-based theories, this model predicts that priming should occur from attended primes regardless of whether they are familiar or unfamiliar views of objects, but should only occur from unattended primes that are familiar views; a pattern that has been confirmed behaviourally (Stankiewicz et al., 1998; Thoma et al., 2004).

**Fig. 1:**
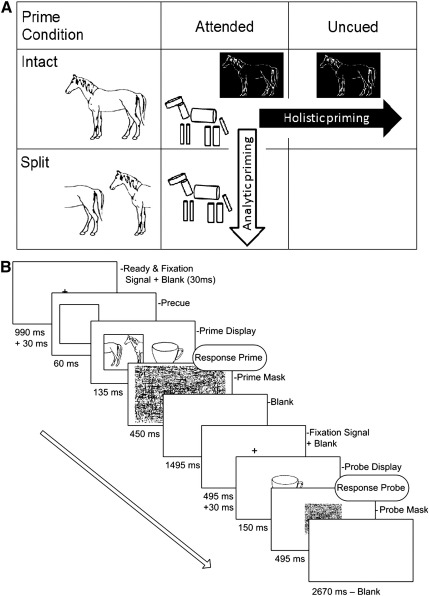
(A). Examples of intact and split images used in the current study and graphic representation of the experimental design. Both attended and uncued objects prime their corresponding probe when shown in an intact configuration (holistic priming, depicted by contrast-reversal), resulting in a main effect of configuration. Both intact as well as split objects prime an intact probe object when attended (analytic priming, depicted as cylinders), resulting in a main effect of attention.(B) Sequence of events within one trial of the main experiment.

The current study therefore investigated the brain regions that support visual object constancy by using fMRI to examine how neural repetition effects relate to this hybrid model. More specifically, we employed an alternative to view transformation by vertically splitting line-drawings of objects. This manipulation has been argued to be a simple means with which to distinguish between part-based and view-based representations (Thoma et al., 2004; Hayward et al., 2010). Intact and split images of an object (e.g. a horse; see Fig. 1A) are completely different holistic representations (different features are bound to different locations in the image, see Hummel, 2001), but retain an almost equivalent structural representation (the same parts are depicted in many of the same spatial relations). Thus splitting object images disrupts view-specific matching but not recognition based on parts (Thoma et al., 2004; Thoma and Davidoff, 2007). There is accumulating evidence for hybrid representations of objects from behavioural studies in healthy individuals (Stankiewicz, et al., 1998; Stankiewicz and Hummel, 2002; Thoma et al., 2004; Thoma and Davidoff 2006; Thoma et al., 2007) and patients with object agnosia (Davidoff and Warrington, 1999). However, it is not yet clear whether these different types of object representations are supported by different brain regions. One proposal by Marsolek (1999) suggests that analytic vs holistic representations are favoured by left and right hemispheres respectively (see also Burgund and Marsolek, 2000).

The second important manipulation of the present experiment was whether or not the object primes were spatially cued in order to manipulate visual attention. According to the hybrid model it is predicted that repetition effects occur for attended intact and split objects, whereas uncued objects show repetition effects only in familiar intact views. A few previous imaging studies have examined the role of attention in visual object repetition effects, but their implications for hybrid models are unclear. For example, two studies found RS in ventral stream regions across mirror-reflected images of objects: one only when those images were spatially cued (Eger et al., 2004), the other even when those images were uncued (Vuilleumier et al., 2005). However, mirror-reflection is arguably suboptimal for dissociating different types of representations, because in many cases it does not prevent overlap of low-level (2D) features (unless the object is highly asymmetrical) and because a mirror view of an object is also likely to be a familiar (learned) view. Such findings therefore do not allow strong conclusions about view constancy or holistic vs part-based representations. Furthermore, in the study that did find RS for uncued objects, cued and uncued objects occupied the same location but in different colours (e.g., Vuilleumier et al., 2005). This feature-based manipulation of attention may not be as effective as spatial cueing in minimising attentional slippage to the uncued stimulus (Eriksen and St James, 1986; Lachter et al., 2004; Henson and Mouchlianitis, 2007).

The present study addressed these issues by examining the BOLD response to an intact object image (the “probe” stimulus) as a function of immediately-preceding presentation of either the same or a different object (the “prime” stimulus), where the prime was manipulated by factorially crossing its configuration (intact vs split) with its cued location (attended vs uncued). The study was also designed to address other methodological details that might have affected previous fMRI studies of visual object priming. First, some previous fMRI studies (Eger et al., 2004) re-used the same objects across trials. “Long-lag” repetition effects across trials (Henson et al., 2004) may have reduced sensitivity to the short-lag, within-trial repetition effects of interest (e.g., by “saturating” effects of repetition). Therefore, every object in the current study was only shown in one trial. Second, many fMRI studies of object priming (Vuilleumier et al., 2002; Eger et al., 2004) employed semantic decision tasks, which may be suboptimal in disentangling perceptual from semantic contributions to repetition effects. For example, post-perceptual, semantic contributions to the BOLD signal may have reduced the differences in RS across their visual transformations (see, e.g., Horner and Henson, in press). In contrast, behavioural priming studies have favoured naming tasks, because the demands on naming are additive with the amount of perceptual change (Biederman and Cooper, 1991; Bruce et al., 2000). Participants in the current study therefore performed a covert naming task; covert in order to minimise fMRI artefacts induced by speech-related movement, but accompanied by a key press to provide an RT measure of priming. Finally, selective attention was manipulated by spatial cueing of briefly-displayed objects, which is likely to minimise the influence of top–down processing (e.g., as compared to colour-based cueing of overlapping figures, Vuilleumier et al., 2005).

Our main focus was on regions within the ventral and dorsal visual-processing streams (Milner and Goodale, 1995). Most previous fMRI studies of visual object recognition have focussed on the ventral stream, with the more anterior regions (e.g., fusiform gyrus) tending to show greater generalisation of RS across low-level visual transformations than more posterior (e.g., occipital) regions (Grill-Spector et al., 1999; Vuilleumier et al., 2002). However, there are also regions within the dorsal stream that respond to visual objects, particularly in parietal cortex, and which can show response increases (repetition enhancement, RE) to primed objects (Dolan et al., 1997; Eger et al., 2007). Regions in the dorsal stream are generally thought to mediate the visuomotor transformations required for control of actions (Goodale and Milner, 2004). Parietal lesions can lead to problems processing objects in “unusual views” (Warrington and Taylor, 1978; Layman and Greene, 1988; Warrington and James, 1988), or with integration of multiple items/objects into a coherent whole (Humphreys et al., 1992). In this case, parietal regions should show greater BOLD signal for split than intact objects (e.g., in our localiser session), and repetition effects in parietal regions should be sensitive to prime configuration.

In summary, the aim of our study was to test the hybrid model of Hummel (2001) that allows for both analytic (part-based) and holistic (view-based) processing. According to this model, brain regions supporting analytic processing should show repetition effects from both intact and split prime objects, but only when attended, whereas brain regions supporting holistic processing should show repetition effects only from intact prime objects, whether attended or not (see Fig. 1A). We expected that analytic processing would involve anterior ventral stream regions (most likely fusiform, Grill-Spector et al., 1999), particularly in the left hemisphere (Marsolek, 1999; Vuilleumier et al., 2002). Holistic processing, on the other hand, was expected to involve more posterior ventral stream regions, and also possibly parietal regions in the dorsal stream, which have been previously been implicated in priming paradigms (Dolan et al., 1997; Eger et al., 2007). A finding that the effects of attention and of configuration on fMRI responses to object repetition occur in different brain regions would provide further, neural support for the hybrid model.

## Materials and methods

### Participants

Twenty-one, neurologically-healthy participants were recruited for this study. Of these, four participants were dropped because they could not covertly name (as indicated by a button press) either the prime or probe on more than one half of the trials (leaving seventeen participants). They were healthy right-handed volunteers (9 males), with a mean age of 26 years (range 19–41) and with normal or corrected vision. The study was approved by a local research ethics committee (LREC reference 05/Q0108/401). The participants were informed they could withdraw from the study at any point and they gave their written consent before participation.

### Stimuli

As in Thoma et al. (2004), we used the manipulation of splitting an image to investigate the nature of visual representations. The rationale is that intact and split images of an object (e.g. a horse) are completely different holistic representations (different features are statically bound to different locations in the image, see Hummel, 2001) whilst they remain highly structurally similar to observers because they depict the same parts in roughly equivalent spatial relations. Splitting an image is unproblematic for the integrity of a structural description as long as the shapes of the object's parts (such as the halves of the horse's torso in Fig. 1A) are recoverable from the information presented in each half of the image (Biederman, 1987; Hummel and Biederman, 1992). The recovery is feasible because for shape recognition the relations between connected parts (e.g., the split half depicting the front of the horse) are more important than relations between separated parts (Saiki and Hummel, 1996). Indeed, Cave and Kosslyn (1993) showed that split stimuli are substantially easier to recognise than scrambled objects that disturb the spatial relationships between parts (for a further account on the rationale for split images, see Thoma et al., 2004).

We used 748 digitised black and white line drawings of familiar objects derived from different sources (Snodgrass and Vanderwart, 1980; Cycowicz et al., 1997). For each object, a “split” version was created by using a 50–60% “offset” filter in Adobe Photoshop 5.5, resulting in images that appeared to be cut vertically in two halves that were relocated to the opposite side of the canvas (see Fig. 1A). The images were standardised in size such that they subtended 4° of visual angle along their main axis of elongation (i.e. horizontal or vertical) when viewed in the MRI scanner. A random-line pattern mask that covered the whole screen (15.6° of visual angle) was presented following prime displays and a smaller random-line pattern mask (4.6° × 3.45°) was presented at the screen centre following probe displays. An outline circle (0.25°) and a fixation cross (0.25°) were presented before each trial.

A prime display consisted of a target object that appeared either 4° left or right of fixation in a cueing square, and an uncued object that appeared at the other side of fixation. The probe display consisted of a single object shown at the centre of the screen. The probe object was equally likely to be the same as the uncued prime, the same as the task-irrelevant prime, or a new image. The left or right location and the configuration of the probed and unprobed object (intact or split) were counterbalanced for each of the 3 repetition conditions (attended repeated, uncued repeated, or no object repeated). These 24 (2 hemifield × 2 configuration probed × 2 configuration unprobed × 3 repetition) variations of prime–probe trial pairs were repeated 11 times constituting 264 trial pairs in total. Each object appeared only in one prime–probe pair throughout the experiment.

The assignment of objects to experimental conditions was controlled across participants by placing each image into one of 8 subsets: 6 subsets with 44 objects that appeared as both prime and probe objects; and 2 subsets containing 176 images that were used as “fillers” for prime objects that never appeared as a probe. The first 6 subsets were counterbalanced across participants so that each object appeared in a particular condition (attended-intact, attended-split, uncued-intact, uncued-split, unprimed-intact, unprimed-split) equally often as a probe. The 2 subsets of “filler objects” in the prime display were randomly assigned as attended (not probed) and uncued (not probed) in both intact and split configurations. The instructions and stimuli were shown on a screen located ~ 90 cm above the participants' head and viewed via a mirror on the head coil.

A separate ‘localiser’ session was run after the main experiment to determine brain regions generally responsive to the object images used. A set of objects different from those in the priming study was presented in addition to ‘scrambled’ versions of 120 objects drawn from the “fillers” in the main experiment. The scrambling was done by first rotating an object and then using a filter in Adobe Photoshop 6.0 which divided the image into 20 quadrants and randomly moved them within the original frame. The reason for using a separate localiser session, rather than using an orthogonal localising contrast within the main experiment, reflected the concern that the inclusion of additional trials with fully-scrambled objects would disrupt the main experimental task, and this would deviate from replicating our prior behavioural studies (see Friston et al., 2006).

### Procedure

The main experiment consisted of two short practise sessions (one outside and one inside the scanner), three experimental sessions (two 12 minute sessions and one 8 min session), with a 1-minute break in between, plus a localiser session (6 min) at the end. Structural scans were taken between experimental and localiser sessions. The whole experiment lasted about 55 min.

Participants were instructed to watch a sequence of trial-pairs consisting of prime and probe displays. They were told to attend to the object in the cued location and name it covertly and press a button with their right index finger as soon as they had named the object. Participants were instructed to ignore the object presented on the uncued side.

The participants read instructions, which they then paraphrased back to the experimenter. The experimental session began with 12 practise trials using a set of images different from the experimental set. After the practise trials, the participants were asked whether they had any questions. Another practise session (with the same images as before) was run inside the scanner. The ordering of the 264 experimental trials and the pairing of attended and uncued objects on prime trials were randomised for each participant.

The basic sequence of events within one trial is depicted in Fig. 1B. An unfilled circle in the centre of the screen remained for 495 ms. The circle was then replaced with a fixation cross, which remained on the screen for another 495 ms, followed by a blank white screen for 30 ms. An attentional cueing square subtending 4.5° of visual angle was then presented either to the left or right of the fixation cross, centred 4.0° from fixation. After 60 ms, two object images were displayed simultaneously for 135 ms, with the attended image inside the square, and the unattended image centred 4.0° from fixation on the other side of the screen. The prime images could be both intact, both split or one of each. After the images disappeared a random-line pattern mask that covered the entire screen (15.6° of visual angle) was shown for 495 ms. The entire prime display lasted less than 200 ms, a duration too short to permit a saccade to the cueing square or either object.

Following the prime display, a blank screen was displayed for 1495 ms, followed by a fixation cross (495 ms). After a 30 ms blank screen, the probe image was displayed in the centre of the screen for 150 ms. The probe object was either the previously attended object (attended conditions), the uncued object (uncued conditions), or an object the participant had not seen previously in the experiment (unprimed baseline condition). The probe image was always intact. The probe display was followed by a single pattern mask (4.6°) shown for 495 ms, which in turn was followed by a blank screen lasting 2670 ms. Again, the participant's task was to name the probe object as quickly and as accurately as possible and press a button. A trial lasted about 7 s and started automatically after the previous trial had ended.

For the localiser session, stimuli were presented in blocks of 3 types: 5 blocks of intact objects, 5 blocks of split objects and 10 blocks of scrambled objects, presented in alternated sequence (intact, scrambled, split, scrambled, etc.). Each block contained 13 trials, presented with a SOA and ISI of 1500 ms and 1000 ms, on which participants made a 1-back judgment (i.e. press the button whether the previous image was the same as the current one, which happened in 1 out of 13 trials on average across the session). There was a 500 ms pause between blocks.

### fMRI acquisition

Thirty-two T2*-weighted transverse slices (64 × 64 3mm × 3mm pixels, TE = 30 ms, flip-angle = 78°) per volume were taken using Echo-Planar Imaging (EPI) on a 3T TIM Trio system (Siemens, Erlangen, Germany), with a repetition time (TR) of 2000 ms per volume. Slices were 3-mm thick with a 0.75 mm gap, tilted approximately 30° at the front to minimise eye-ghosting, and acquired in descending order. The main experiment was split into three sessions, producing 935 functional volumes in total; the localiser session contained 203 scans (excluding the first 10 volumes per session in both cases, to allow for spin equilibration). A T1-weighted structural volume was also acquired for each participant with 1mm × 1mm × 1mm voxels using MPRAGE and GRAPPA parallel imaging (flip-angle = 9o; TE = 2.00 s; acceleration factor = 2).

Data were analysed using Statistical Parametric Mapping (SPM5, http://www.fil.ion.ucl.ac.uk/spm5.html). Preprocessing of image volumes included spatial realignment to correct for movement, followed by spatial normalisation to Talairach space, using the linear and nonlinear normalisation parameters estimated from warping each participant's structural image to a T1-weighted average template image from the Montreal Neurological Institute (MNI). These re-sampled images (voxel size 3 × 3 × 3 mm) were smoothed spatially by a 10 mm FWHM Gaussian kernel (final smoothness approximately 14 × 14 × 14 mm). Note that this smoothing may attenuate signals at a finer spatial scale (e.g., regions with different retinotopy), but will also increase sensitivity to signals at a comparable spatial scale, and is common in group analyses to allow for residual individual differences in anatomy after spatial normalisation, and to fulfil the assumptions behind random field theory, particularly with low degrees of freedom (Nichols and Hayasaka, 2003).

Statistical analysis was performed in a two-level approximation to a Mixed Effects model. In the first level, neural activity to each prime and probe event within a trial was modelled by a delta function at stimulus onset. The BOLD response was modelled by a convolution of these delta functions by a canonical Haemodynamic Response Function (HRF). The resulting time-courses were down-sampled at the midpoint of each scan to form regressors in a General Linear Model (GLM).

For each main experimental session in the GLM, 11 separate regressors were modelled: 7 locked to probe onset and 4 locked to prime onset. The probe-locked events consisted of 6 trial-types of interest, plus a 7th trial-type to model error trials of no interest (errors were trials in which participants did not press a key for either the prime or probe; see Behavioural results). The 6 trial-types of interest were attended-intact, attended-split, uncued-intact, uncued-split, unprimed-intact and unprimed-split. The 4 prime-locked events were differentiated according to whether the attended prime was intact or split, and on the left or right of fixation. Note that these regressors were not of interest, but included in the GLM so as to remove effects on the BOLD response locked to the probe (which followed shortly after) that were caused by the nature of the prime. Because these prime events were not split according to the primed vs unprimed nature of the probe, their inclusion did not affect differences between primed and unprimed probe-locked responses. To account for (linear) residual artefacts after realignment, each session also included six further regressors representing the movement parameters estimated during realignment. Voxel-wise parameter estimates for these regressors were obtained by Restricted Maximum-Likelihood (ReML) estimation, using a temporal high-pass filter (cut-off 128 s) to remove low-frequency drifts, and modelling temporal autocorrelation across scans with an AR(1) process.

The localiser data were fit using the same type of linear-convolution GLM, which contained two regressor modelling epochs of 20 s duration: one for intact object blocks and one for split object blocks (scrambled object blocks thus comprising the implicit baseline).

Four “repetition” contrasts were evaluated on the parameter estimates estimated in the main experiment GLM, in which the unprimed-intact and unprimed-split conditions were subtracted from the corresponding attended and uncued primed conditions (averaging across sessions).

We performed two types of analysis on these repetition contrasts: 1) a voxel-wise analysis on normalised images, and 2) a functionally-defined region-of-interest (fROI) analysis on a handful of regions defined from the independent localiser data. The first analysis offers an exhaustive search for effects across the brain (in case effects are found beyond a priori regions of interest); the second analysis allows for factorial analyses across brain regions (e.g., by adding factors of left/right, anterior/posterior), where those regions are defined independently of the condition effects of interest (see Results).

### Voxel-wise analyses

Images of the four repetition contrasts comprised the data for a second-level model, which treated participants as a random effect, corresponding to a 2 × 2 (Attention × Configuration) repeated-measures ANOVA on repetition effects (analogous to the ANOVA performed on the behavioural data). Within the second-level model, Statistical Parametric Maps (SPMs) were created of the T or F-statistic for the various ANOVA effects of interest, using a single pooled error estimate for all contrasts, whose nonsphericity was estimated using ReML as described in Friston et al. (2002). For the localiser fMRI data, separate second-level models (conforming to one-sample T-tests) were performed for first-level contrasts of 1) the average of intact and split objects vs Scrambled objects and 2) intact vs split objects. Unless otherwise stated, the SPMs for repetition effects were height-thresholded at the voxel-level at p < .05, corrected for multiple comparisons using Random Field Theory for mask images containing voxels that showed greater activity for intact than Scrambled images, or for split than intact images, in the corresponding second-level models of the localiser data. Stereotactic coordinates of the maxima within the thresholded SPMs correspond to the MNI template.

### Group-fROI analyses

In this analysis, the contrast values for the same repetition contrasts described in the first-level analyses above were extracted from spherical volumes of 8 mm radius that were centred on selected maxima defined independently by the localiser contrasts of intact, split and scrambled objects (as has been common in neuroimaging studies of visual object recognition; e.g., Saxe et al., 2006). These fROI repetition effects were subjected to the same repeated-measures ANOVA as the behavioural data, but with additional factors of Laterality (left vs right) and, for the ventral stream, Rostrality (anterior vs posterior), in order to address hypotheses raised in the Introduction.

## Results

### Behavioural results

#### Prime responses

Trials in which participants did not press a button to primes, or in which button presses to primes were faster than 300 ms, were counted as errors. One-way within-participant analyses of variance (ANOVAs) were conducted on the correct RTs and error rates for the attended prime object, with the factor of configuration (split, intact). These ANOVAs showed a main effect on the RTs, F(1, 16) = 10.3, p < .01, MSE = 2485, and on the error rates, F(1, 16) = 19.2, p < .001, MSE = 2.64 . Mean RTs were 847 ms for intact images (M error rate = 13%) and 899 ms (M error rate = 24%) for split images. Thus, configuration was effectively manipulated in the processing of the prime display, with poorer performance for split objects, as previously observed by Thoma et al. (2004). This indicates that participants complied with instructions and named objects subvocally.

#### Probe responses

Trials with latencies longer than 3000 ms or shorter than 300 ms were counted as an error (M = 8%). In all conditions, priming was calculated as the participant's mean RT at probe in the unprimed (baseline) condition minus their mean RT in the corresponding experimental condition. Trials on which either the prime or probe responses were errors (M = 20%) were excluded from the statistical analysis.

A 2 (Attention: attended vs. uncued) × 2 (Prime Configuration: intact vs. split) within-participants analysis of variance (ANOVA) revealed a reliable main effect of Attention, F(1, 16) = 22.0, p < .001, and a marginal effect of Prime Configuration, F(1, 16) = 3.79, p = .07. The interaction between Attention and Prime Configuration did not approach significance, F(1, 16) < 1 (see Table 1). There were no indications of a speed-accuracy trade-off in any condition.

**Fig. 2:**
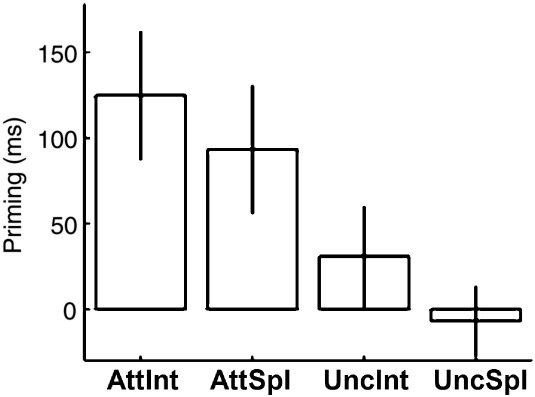
Behavioural priming, i.e., unprimed–primed RTs, for each condition of interest (AttInt = attended, intact condition, AttSpl = attended, split condition, UncIn = uncued, intact condition, UncSpl = uncued, split condition). Error bars reflect one-tailed 95% confidence intervals.

Analysis of each priming condition was performed to determine which type of prime display caused savings in response time for the probe display (i.e., faster naming responses relative to unprimed probes) using one-tailed t-tests. Priming was reliably greater than zero in the attended-intact, t (16) = 5.51, p < .001; attended-split, t (16) = 4.19, p < .001; and uncued-intact, t (16) = 2.20, p < .05, conditions, but not in the uncued-split condition, t (16) < 1 (see Fig. 2). This replicates earlier behavioural results obtained with overt naming tasks (Thoma et al., 2004), and shows that split images prime an intact probe image only when attended, but not when uncued, whilst intact images prime themselves when attended as well as uncued. Indeed, it is noteworthy that the RTs for a button press associated with covert naming in the scanner are sensitive enough to replicate the pattern of behavioural priming effects.

#### Localiser responses

The only dependent variable of interest here was accuracy, which was 93%, indicating that participants adhered to instructions and performed well.

## Imaging results

### Voxel-wise analysis — Localiser results

Our interest was in BOLD repetition effects in brain regions showing significant responses to objects. To define these brain areas sensitive to line-drawings of familiar objects, we used a localiser block for each participant in which objects were shown in an intact configuration, split into two halves, or fully scrambled (see Materials and methods).

**Fig. 3:**
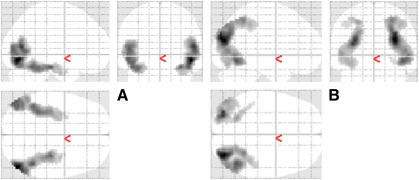
Maximal intensity projections (MIP) of clusters that survived p < .05 corrected for their spatial extent (using a height threshold of p < .001 uncorrected) in the localiser session for the contrasts of: (A) objects (averaging over intact and split) vs scrambled objects, and (B) split vs intact objects.

Contrasting the BOLD response to objects (averaging across intact and split) vs scrambled objects, we obtained greater responses to objects in expected bilateral ventral visual stream regions, from lateral occipital to anterior ventral temporal cortex (see Fig. 3A and Supplementary Table 1).

We also contrasted BOLD responses for intact vs split objects. No voxels showed greater responses to intact objects at the corrected threshold, but two reliable bilateral clusters of voxels were found to show greater responses for split images, which extended along the dorsal visual stream, from dorsal occipital lobes to bilateral superior parietal gyri and the intraparietal sulcus (see Fig. 3B and Supplementary Table 2). These response increases may correspond to visuospatial, mental imagery processes by which participants “fused” the two halves of split objects.

In summary, the two orthogonal localiser contrasts of objects vs scrambled objects, and of split objects vs intact objects, elegantly revealed ventral and dorsal visual processing streams respectively (cf. left and right panels of Fig. 3).

### Voxel-wise analysis — Repetition effects

Our primary interest was in BOLD repetition-suppression (RS) and repetition enhancement (RE) effects in brain regions that respond to visual objects (split or intact). Thus we report effects of repetition that survived p < .05, two-tailed, corrected for the whole brain, or for search volumes defined by either the above two localiser contrasts. (For additional voxels that survived a similar threshold but outside the localiser masks, see Supplementary Table 3.)

As with the behavioural ANOVA, we tested three effects of Attention and Configuration on repetition effects: the main effect of Attention, main effect of Configuration and their interaction. These are reported in Table 2. According to hybrid theories of object recognition, analytic representations should be revealed by the main effect of attention (more specifically, RS is expected for attended-intact and attended-split conditions, but not for uncued-intact or uncued-split conditions), whereas holistic representations should be revealed by the main effect of configuration (more specifically, RS is expected for attended-intact and uncued-intact conditions, but not attended-split or uncued-split conditions). The interaction is not necessarily predicted by hybrid models, but could be explained if the effects of holistic and analytical representations are super-additive (or by other theories, e.g., that RS only occurs for repetition of identical or visually-similar images that are attended) and is therefore also of interest.

#### The main effect of attention on repetition effects

**Fig. 4:**
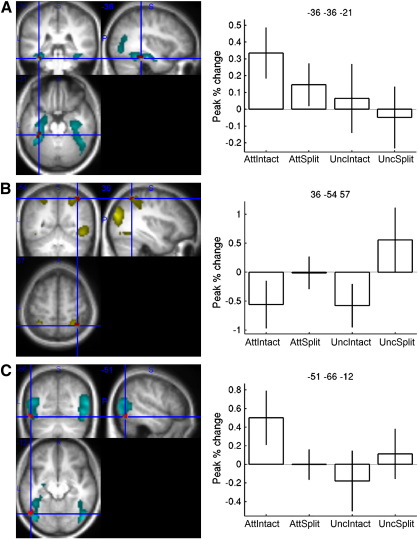
Voxel-wise results. On the left, statistical parametric maps of regions implicated in object recognition shown on orthogonal sections through a normalised structural of one participant. (A) Voxels in cyan were more active for objects than scrambled objects in the localiser (Fig. 3), and formed the search space for the voxels in red that showed a main effect of attention on repetition effects in the main experiment, thresholded at p < .001 uncorrected. Cross-hair centred on the left fusiform maximum that was the only maximum to survive correction for the search region. (B) Voxels in yellow were more active for split than intact objects in the localiser (Fig. 3), and formed the search space for the voxels in red that showed a main effect of configuration on repetition effects in the main experiment, thresholded at p < .001 uncorrected. Cross-hair centred on the right intraparietal maximum that was the only maximum to survive correction for the search region. (C) Voxels in cyan were more active for objects than scrambled objects in the localiser (Fig. 3), and formed the search space for the voxels in red that showed an interaction between attention and configuration on repetition effects in the main experiment, thresholded at p < .001 uncorrected. Cross-hair centred on the left lateral occipital maximum that was the only maximum to survive correction for the search region. On the right, percentage BOLD signal change between the peak of the fitted event-related response to the primed conditions subtracted from that of the corresponding unprimed conditions (i.e., where positive = repetition suppression (RS), and where percentage is relative to the mean BOLD signal over all voxels and volumes), for each of the four conditions of interest (AttIntact = attended, intact condition, AttSplit = attended, split condition, UncIntact = uncued, intact condition, UncSplit = uncued, split condition). Error bars are two-tailed 95% confidence intervals of repetition effects vs zero.

An F-contrast for the main effect of attended vs uncued conditions on RS (averaged across intact and split primes) revealed only one maximum in left anterior fusiform (Fig. 4A) that survived correction for the search region defined by object-responsive voxels in the localiser contrast of objects vs scrambled objects (no maxima survived correction for the localiser contrast of split vs intact objects). This fusiform maximum showed RS from both attended conditions (Fig. 4A), though larger RS from attended-intact than attended-split conditions (post hoc T(16) = 2.16, p < .05, two-tailed).

#### The main effect of configuration on repetition effects

An F-contrast for the main effect of intact vs split configuration on repetition effects (averaged across attended and uncued primes) did not reveal any maxima that survived p < .05, two-tailed corrected for either of the localiser contrasts. However, there was a maximum in right intraparietal sulcus (Fig. 4B) that survived p < .10 corrected for the localiser contrast of split vs intact objects. This maximum showed RE (larger responses from primed than unprimed) for intact (but not split) conditions (Fig. 4B). Given prior evidence that parietal cortex shows RE during similar paradigms, such that one could make a one-tailed prediction for this region, plus further evidence for RE in the fROI analyses below, we considered this voxel-wise effect worth reporting.

#### Interaction of attention and configuration on repetition effects

Finally, an F-contrast for the interaction between attention and configuration on repetition effects revealed only one maximum in left lateral occipital cortex (Fig. 4C) that survived correction for the object-responsive voxels in the objects vs scrambled objects localiser contrast (no maxima survived correction for the localiser contrast of split vs intact objects). (For voxels showing reliable simple effects of repetition within each condition, see Supplementary Table 4.)

Note that none of the above effects of attention and/or configuration occurred within voxels that showed a reliable difference between whether cued primes were left or right of fixation (see Supplementary Fig. 1), suggesting that they do not show strong retinotopy (at least at the level of attended visual field).

#### Group-fROI analysis — Ventral visual stream

Given the hypotheses outlined in the Introduction about functional specialisation along the posterior–anterior and left–right axes of the ventral stream, we investigated BOLD repetition effects across four fROIs in the ventral stream. To define these fROIs independently of the above voxel-wise repetition effects, the data for the main experiment were extracted from volumes centred on the maximum of the contrast of objects vs scrambled objects in the independent localiser data in Supplementary Table 1 – specifically the left and right inferior temporal gyrus maxima ([− 51 − 75 − 6] and [+ 48 − 72 − 5]) – henceforth, “posterior” ventral stream fROIs – and left and right mid fusiform gyrus maxima ([− 42 − 48 − 18] and [45 − 51 − 18]) – henceforth, “anterior” ventral stream fROIs. Analogous results when the coordinates of these fROIs were defined individually from the SPM for each participant's localiser data, rather than the present group-based SPM, are shown in the Supplementary Materials.

**Fig. 5:**
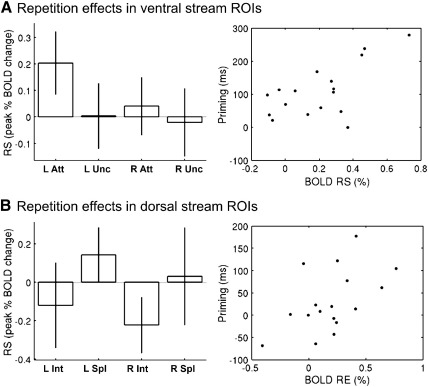
Group-fROI results (A) On the left, BOLD repetition effects for attended and uncued conditions (averaged across intact and split configurations) for left (L) and right (R) fROIs within the ventral visual stream (averaged across posterior and anterior fROIs; see text). See Fig. 4 legend for further details. On the right, scatter plot of each participant's behavioural priming for attended primes (averaged across intact and split configurations) against RS in left fROI regions (averaged across posterior and anterior fROIs). (B) On the left, BOLD repetition effects for intact and split configuration conditions (averaged across attended and uncued) for left (L) and right (R) fROIs within the dorsal visual stream (see text). On the right, scatter plot of each participant's behavioural priming against RE for uncued, intact primes in intraparietal fROIs (averaged across left and right fROIs).

A 2 × 2 × 2 × 2 ANOVA on BOLD repetition effects, formed by adding the factors of “Laterality” and “Rostrality”, revealed a highly reliable interaction between Attention and Laterality, F(1, 16) = 22.4, p < .001 (in addition to a main effect of Laterality, F(1, 16) = 11.2, p < .005, and the expected main effect of Attention, F(1, 16) = 5.00, p < .05). A plot of the mean repetition effects corresponding to this interaction (i.e., averaging across the factors of Configuration and Rostrality) showed reliable RS in attended conditions in the left hemisphere, but not in the right (Fig. 5A). This was confirmed by separate ANOVAs on left and right hemisphere fROIs, which showed a reliable main effect of Attention in the left, F(1, 16) = 10.5, p < .005, but not in the right, F(1, 16) = 1.10, p = .31, hemisphere. Furthermore, the amount of priming showed a positive correlation across participants with the amount of RS for attended objects averaged across the left hemisphere fROIs (and across Rostrality and Configuration), Pearson's R = 0.64, p < .01 (Fig. 5A, right panel). There was no such correlation for right hemisphere fROIs, or for uncued conditions in either left or right fROIs, Rs < .29, ps > .27.

No other interactions reached significance in the 2 × 2 × 2 × 2 ANOVA, except the three-way interaction between Rostrality, Laterality and Configuration, F(1, 16) = 10.1, p < .01. However, in separate ANOVAs on left and right fROIs (averaged across Attention), no effects of Rostrality or Configuration reached significance in either hemisphere, Fs < 3.15, p > .09, so this three-way interaction was not explored further.

#### Group-fROI analysis — Dorsal visual stream

Given the hypotheses outlined in the Introduction about the role of the dorsal visual stream in visual object recognition, and the effect of configuration on repetition effects in the right intraparietal sulcus (IPS) in the above voxel-wise analysis, we further investigated BOLD repetition effects in fROIs defined by the contrast of split vs intact objects in the independent localiser data — specifically the left and right IPS maxima in Supplementary Table 2. Analogous results for individually-defined fROI coordinates are shown in the Supplementary Materials. A 2 × 2 × 2 ANOVA with factors Laterality, Attention and Configuration revealed only a main effect of Configuration, F(1, 16) = 4.48, p < .05, and a main effect of Laterality, F(1, 16) = 9.13, p < .01. A plot of the mean repetition effects (averaging across Attention) showed reliable repetition enhancement in the intact conditions (Fig. 5B), at least in the right intraparietal sulcus (consistent with Fig. 4B). There was no reliable correlation between the amount of priming and the size of the BOLD repetition effect from intact, R = .17, p = .50, or split, R = .15, p = .58, objects when averaging across Attention (and Laterality). The absence of such a relationship would nonetheless be expected if priming in the attended conditions was determined primarily by RS in the ventral stream, as suggested by Fig. 5A.^1^ Thus when analysing uncued conditions only, there was now a reliable positive correlation between priming and the amount of RE from intact objects, R = .52, p < .05 (Fig. 5B, right panel), as expected, but not from split objects, R = −.23, p = .37 (where there was no net priming).

## Discussion

The present study is the first to provide neural evidence for the hybrid model of visual object recognition (Hummel, 2001). As predicted by this model, attended objects showed fMRI repetition effects for both intact and split views when attended, but repetition effects were strictly view-based for unattended objects. Behavioural priming effects in the form of faster covert naming were also observed from attended primes in both intact and split configurations, but only from uncued primes in an intact configuration, replicating previous findings (e.g., Thoma et al., 2004). This pattern was evident in two main effects (more priming from attended than uncued primes, and more priming from intact than split primes), with no interaction, which is consistent with the operation of two parallel routes: an analytic route in which part-based representations can generalise across split images, but which requires attention, and a holistic route in which view-based representations can be accessed without attention (Hummel, 2001). Our fMRI data suggest that these two routes map broadly onto ventral and dorsal visual streams respectively.

Importantly, the fMRI and behavioural repetition effects were inter-related: Firstly, the amount of repetition suppression (RS) in the left ventral stream (averaged across “posterior” and “anterior” functionally-defined regions of interest, fROIs) correlated positively with the amount of behavioural priming, but only for attended primes, as would be expected from contributions from an analytical pathway.^2^ Secondly, the amount of repetition enhancement (RE) in the dorsal stream (averaged across left and right fROIs) correlated positively with the amount of behavioural priming, but only from uncued, intact primes, as would be expected from contributions from a holistic pathway. Below, we review these aspects of the present fMRI findings in relation to previous studies, before discussing the implications of the present findings for theories of visual object recognition.

### Comparison with previous fMRI studies of object repetition effects in ventral stream

One reason for the current investigation was that the manipulation of viewpoint in most previous fMRI studies does not always allow strong conclusions about the types of representations involved in object recognition, because most object recognition theories include some form of view-specific representation (Biederman, 2000), or predict some viewpoint-dependent effects even for part-based representations, (Hummel and Biederman, 1992; Thoma and Davidoff, 2006). Thus so-called “view-dependent” effects based on manipulations of object orientation may not reliably indicate whether the neural populations involved in RS are truly view-based rather than part-based. In contrast, the RS observed here using split images cannot be explained by view-interpolation, because a split image is by definition not a familiar view.

A number of previous fMRI studies have suggested a greater degree of abstraction from posterior to anterior regions along the ventral visual stream, for example between posterior and anterior parts of the ventral visual stream (e.g., Grill-Spector et al., 1999; James et al., 2002). Our fROI analysis, based on regions defined by our localiser contrast of objects vs scrambled objects, did not reveal any difference between posterior (inferior temporal) and anterior (mid-fusiform) fROIs in the effects of intact vs split configurations on the size of RS (it only revealed a main effect of attention on RS, averaging across these two regions). Nonetheless, it is interesting to note that the maxima identified in our voxel-wise analysis showed reliable RS from both intact and split attended primes in an anterior fusiform region (Fig. 4A), but RS only from intact, attended primes in a lateral occipital region (Fig. 4C), which is consistent with prior evidence for some degree of view-independence in fusiform cortex (e.g., Koutstaal et al., 2001; Vuilleumier et al., 2002; Simons et al., 2003; Eger et al., 2004). Further experiments are needed to provide more definitive data on this question of abstraction along the posterior–anterior axis of the ventral stream.

Furthermore, both anterior fusiform and lateral occipital maxima identified by the voxel-wise analysis showed greater RS from intact than split images, when attended (which appeared to drive the interaction between configuration and attention on RS for the lateral occipital region, and which was reliable in an unbiased, post hoc *t*-test for the anterior fusiform region). This is consistent with the general pattern in previous studies, that RS is greatest when both prime and probe stimuli are shown in the same rather than a changed view (Grill-Spector et al., 1999; Gauthier et al., 2002; Ewbank et al., 2005; Fang et al., 2006; Andresen et al., 2009). Thus, when these voxel-wise results are considered in conjunction with the fROI results, it seems likely that the left ventral stream actually contains a combination of view-specific as well as part-based representations of object shape. This interpretation is to some degree at odds with claims from a recent study by Hayworth and Biederman (2006) in which RS in lateral occipital cortex was attributed almost exclusively to the repetition of part-critical vertices, and only to a lesser degree to the repetition of local contours (see Biederman and Cooper, 1991). However, stimulus pairs in these experiments were apparently repeated a number of times, making it difficult to assess the part-based contribution from one-shot recognition. Furthermore, RS was measured using a long-lag priming paradigm with several minutes between prime and probe stimulus, which is known to produce view-invariant effects that disappear or are attenuated when a short-lag paradigm is used (Epstein et al., 2008; Andresen et al., 2009). Overall, our results suggest that the neural correlates of object repetition in the ventral stream are more dependent on the holistic shape of an object than implied by Hayworth and Biederman's findings.^3^

We did not find any repetition effects in the ventral stream for uncued objects. This is contrary to some previous studies (e.g., Murray and Wojciulik, 2003; Vuilleumier et al., 2005), but consistent with others (e.g., Eger et al., 2004; Henson and Mouchlianitis, 2007). One reason for this difference may be that we used a spatial manipulation of attention, rather than a feature- or object-based manipulation of attention to spatially-coincident stimuli (Murray and Wojciulik, 2003; Vuilleumier et al., 2005) which may not have fully prevented participants from attending uncued stimuli (Eriksen and St James, 1986; Lachter et al., 2004). In this case, it would appear that repetition effects in the ventral stream depend strongly on attention (see Henson and Mouchlianitis, 2007, for further discussion). Nonetheless, the present study did find reliable repetition effects in the dorsal stream from uncued intact primes, which is discussed next.

### Object-related activity in the dorsal stream

The voxel-wise analyses showed bilateral regions within the dorsal stream that were more active for split than intact objects in the localiser session (Fig. 3B), within which a maximum in the right intraparietal sulcus (IPS) showed RE from intact primes in the main experiment, regardless of attention (Fig. 4B). The fROI analysis confirmed reliable RE in the right IPS from intact primes, which correlated with the amount of behavioural priming in the intact, uncued condition. These findings are generally consistent for a role of the dorsal stream in visual object processing (see Introduction), though the nature of this processing deserves further consideration.

One explanation for the increased BOLD response to split than intact images in the localiser is that IPS supports visual transformations that are needed to identify split stimuli (see Introduction). In this case, one might expect RS in this region when a split probe is primed by a split (or intact) prime, owing to prior facilitation of these transformations. Note, however, that the split condition in the present paradigm refers to the prime, and that the probe was always an intact image. Thus whilst this explanation might account for the IPS BOLD increases in the localiser session, it is not clear that it accounts for the RE from intact but not split primes in the main experiment.

An alternative explanation of the RE in IPS is that it reflected a “match” with the contents of visual short term memory (VSTM) (Todd and Marois, 2004; Xu and Chun, 2006). Assuming these contents were image-based representations, then a match would only occur for intact stimuli.^4^ Nonetheless, there are reasons why the present parietal RE effects are likely to reflect more than simple matching in VSTM. Firstly, the amount of RE in IPS correlated with the amount of behavioural priming in the uncued-intact condition. If this RE in IPS is causally related to behavioural priming, then a pure VSTM matching explanation cannot explain other behavioural priming effects, such as the finding that intact uncued objects do not prime themselves when they are shown upside down in both prime and probe displays (Thoma et al., 2007). Secondly, a previous fMRI study showed that the dorsal stream distinguished between object classes, without any obvious differences in VSTM, suggesting a capacity for object recognition in parietal cortex (Fang and He, 2005).

RE in parietal cortex has been found in at least two other fMRI studies of visual object priming (Dolan et al., 1997; Eger et al., 2007). Although both studies differed methodologically from the present paradigm, their results overall reinforce an important role of parietal cortex in visual object priming. The findings most directly related to the present view-specific repetition effects in IPS come from a study by James et al. (2002). These authors found fMRI response reductions in both lateral occipital cortex and caudal IPS during blocks in which objects were repeated multiple times, but these reductions only generalised over depth rotations in lateral occipital cortex. Thus, IPS responded to repeated (intact) objects only when shown in the same view, as in our study, though this response was RS rather than the RE, unlike in our study. This discrepancy between RS and RE may relate to the differences between two paradigms: The James et al. paradigm involved only passive viewing, and objects were repeated many times within a block (more typical of an “fMR adaptation” paradigm; e.g., Grill-Spector et al., 1999), such that their RS reflected a reduction in the mean response throughout a block. This reduced average response may be caused by reduced attentional demands across the block, as participants begin to expect the same visual image on every trial. Nonetheless, despite the different direction of repetition effects, James et al's conclusion that the dorsal stream codes for object identity in a strict view-based fashion is consistent with our proposal here that the IPS maintains holistic object representations. Indeed, a metrically-veridical representation (rather than an abstract part-based one) would make sense for guiding actions via the dorsal stream (see Introduction; Milner and Goodale, 1995). Thus, although the exact nature of RS vs RE in parietal cortex is difficult to establish, it is clear that they are view-specific and correlate with behavioural priming, suggesting a role in visual object recognition.

### Laterality effects within ventral and dorsal streams

The fROI analysis revealed a clear effect of laterality on RS within the ventral visual stream, whereby only regions on the left showed RS for attended primes. This is consistent with the hypothesis of dissociable neural subsystems (DNS) within left and right hemispheres (Marsolek, 1999): An abstract-category recognition system is assumed to be dominant in the left hemisphere, with the ability to represent features of objects independently, such as non-accidental properties (Lowe, 1985; Biederman, 1987). This subsystem permits the visual system to generalise across different members of a category, given that they usually share a common subset of features. In contrast, the specific-exemplar subsystem (dominant in the right hemisphere) processes object shape as a whole; i.e., features are not represented independently of each other. It is thus sensitive to object shape, and maps different shape exemplars to different output representations. The present data therefore support this general proposal (see also Dien, 2009, for a review on hemispheric differences in object recognition).

A different – though not incompatible – explanation of our laterality effects is that our task involved object naming, which is usually associated with left hemispheric activation. Indeed, the present RS effects, particularly from split objects, could result from simple (covert) name repetition. This would seem unlikely though, given that Chouinard et al. (2008) found that repeating a different exemplar of the same name produced no detectable RS in ventral stream regions (see also Horner and Henson, in press). Nonetheless, the pattern of RS in visual responsive areas is likely to depend not merely on stimulus repetition, but also the task performed on each presentation (Henson 2003). As noted earlier, previous imaging studies have tended to use semantic decision tasks, or at least highly nameable objects, such that their greater RS in the left than right hemisphere (Koutstaal et al., 2001; Vuilleumier et al., 2002; Simons et al., 2003; Eger et al., 2004; Zago et al., 2005; Ganel et al., 2006) could also reflect covert naming. Evidence for the role of task demands comes from a review of laterality studies (Grabowska and Nowicka, 1996): For tasks that involve active recognition - in particular of high spatial frequency stimuli like our line drawings - the LH shows an advantage, whereas less demanding perceptual tasks typically show no lateralisation or a RH advantage. Furthermore, whilst other studies have found repetition effects in the right hemisphere (albeit still smaller than in LH) our results may reflect a smaller effect size than is typical, due to the shorter presentation time of prime and probe objects (<=150 ms) than in previous studies (see Zago et al., 2005).

There was also a laterality effect on RE in the dorsal stream, with greater RE in right than left IPS. However, this main effect of laterality did not interact with intact vs split primes, so is difficult to relate, for example, to a possible right hemisphere advantage in coding holistic representations. We therefore leave this finding for future exploration.

To summarise the current fMRI effects in conjunction with behavioural priming – and their dependence on experimental parameters to allow comparisons with other studies – we propose the following: In short-lag repetition paradigms – in which objects are not repeated across trials and a naming task is used for both prime and probe objects – reliable RS effects for same and different (non-overlapping) views of spatially-cued objects are observed in left ventral stream regions, with view-specific effects still dominant, in particular in posterior ventral regions. At the same time, view-specific repetition enhancement effects in dorsal stream regions such as intraparietal cortex appear independent of spatial cueing, and mirror behavioural priming.

### Implications for object recognition

The observation that RS effects in the ventral stream are sensitive to the precise view (configuration) seems at first to concur with theories of object recognition that explain object constancy with transformations or interpolations across 2D views of an object (Ullman, 1989, 1998; Poggio and Edelman, 1990; Bulthoff and Edelman, 1992; Logothetis et al., 1994; Tarr, 1995; Tarr and Gauthier, 1998) or by a distributed neural representation across view-tuned neuronal ensembles (Perrett et al., 1998; see Peissig and Tarr, 2007, for a review). However, the concept of view-dependency is not unique to these models and structural description theories also predict certain view-specific effects (Biederman and Gerhardstein, 1993; Biederman, 2000). More importantly, the present evidence for RS from split images cannot easily be accounted for by view-interpolation or view-transformation accounts. A split image of a horse is by definition not a view (in the sense of a holistic representation, see Thoma et al., 2004), in particular if it has never been seen (and encoded) in such a configuration. This has also been established in behavioural work: Thoma et al. (2004) found priming for uncued objects in familiar views, but not for split images, even if both prime and probe images of the same object were split. Other recent work also casts doubt on the idea of view-transformation processes: Even the robust rotation-dependent RS effects obtained by Andresen et al. (2009) showed that visual angle alone was not a sufficient explanatory factor for lateral occipital responses, as would have been predicted by many view-based theories of object constancy.

To account for these and related properties of object recognition without postulating part-based object processing, some recent view-based models (e.g., Edelman and Intrator, 2000, 2003; Ullman, 2007) have proposed templates for object “fragments” instead of templates for whole objects. The fragments are pictorial features that represent the image appearance of object components, unlike genuine 3D volumes postulated by structural description theories. Although the “fragment” approach seems to provide an alternative solution to account for object constancy, experimental evidence for it is inconclusive (Newell et al., 2005). Importantly, fragments derived from learned views (e.g., Edelman and Intrator, 2000, 2003) arguably do not predict visual priming from split images to their intact counterparts because the proposed sets of fragments are tied to specific locations in the visual field (a mechanism called “what + where” coding; see Edelman and Intrator, 2000, 2003). Since the two sets of fragments in the present split and intact images are completely non-overlapping, and prime and probe objects appear in different locations, the fragment model would hardly predict priming from one image to the other (see Hummel, 2003; Thoma et al., 2004). Furthermore, Edelman and Intrator (2003) postulate that multiple fixations are needed during initial encoding in order to establish the various location-dependent object fragments. In the current study, split images primed subsequent intact probe images even though their presentation was too short to permit saccades. Finally, feature-based models such as Ullman's (2007) postulate that repetition effects should be more similar the more two objects (or their images) share informative features. Since both split and intact images share essentially the same informative features, a feature approach alone cannot explain why there is such dominance for intact objects (see Hummel, 2003, for a discussion of fragment and feature accounts).

The present evidence for RS from split objects in left ventral stream (when attended) provides limited support for part-based representations, such as structural descriptions (e.g. Hummel and Biederman, 1992). At the same time, the greater RS in fusiform/inferior temporal areas from intact than split configurations indicates a further, view-specific component. This conclusion could be challenged from a structural description perspective with the argument that splitting an object image may disrupt spatial relations between some parts, resulting in less RS for the subsequent intact version. However, there is evidence that such disruptions are unlikely or minimal (Thoma et al., 2004) and that view-change or splitting reduces priming to the same degree (Thoma and Davidoff, 2007). A further possible objection related to the splitting manipulation is that split images simply require additional processing resources, which reduce any subsequent priming. However, Thoma et al. (2004, Experiment 3) showed that a split (attended) object primed its identical split self just as much as intact images prime each other. Thus, our conclusion is that strictly part-based (structural) accounts of object recognition alone cannot explain the view-specific RS effects.

Rather, we propose that the current results constitute some of the first neural support for a hybrid account of object recognition, which predicts the involvement of both holistic (automatic and view-specific) and analytic (part-based) representations in object recognition (e.g., Hummel, 2001). As would be predicted by a hybrid analytic/holistic account, RS generalised over configurational changes in (left) fusiform areas for attended objects only. The finding that behavioural priming from intact objects was greater than from split objects is also consistent with Hummel's account, in that priming for previously attended objects in the identical (intact) view receives contributions from both analytic representations and holistic (view-based) representations, whereas priming for attended objects in novel (here: split) views relies on analytic representations only. Indeed, in its computational instantiation ("JIM.3", Hummel, 2001), analytic (attention-dependent) and holistic (view-specific) priming components are additive, a prediction that has been confirmed in behavioural work (Stankiewicz et al., 1998; Thoma et al., 2004, 2007; Thoma and Davidoff, 2006) and in the present behavioural data. The finding that RS in the left ventral stream regions was also greater from intact than from split primes, however, suggests that there may be additional view-dependent representations in the ventral stream (or that its activity reflects interactions with activation of holistic representations in the dorsal stream^5^).

## Conclusions

The current findings support hybrid models of visual object recognition that include both analytic and holistic pathways, with the analytic pathway dependent on visual attention. Regions in the left ventral visual stream only showed repetition suppression (RS) from spatially-cued primes, which occurred even for split primes in more anterior fusiform regions, and the amount of this RS correlated with the amount of behavioural priming, consistent with an analytic pathway. Regions in the dorsal stream on the other hand, specifically the intraparietal sulcus, showed repetition enhancement (RE) only for intact primes, regardless of attention, and the amount of RE correlated with the amount of behavioural priming from uncued, intact primes, consistent with a holistic pathway. Nonetheless, the ventral stream regions also showed greater RS from intact than split primes, which would not be expected if these regions utilised purely structural representations, and which deserves further exploration in future neuroimaging studies of visual object recognition.

## Commercial relationships

None.

## Figures and Tables

**Table 1:** Mean response times (ms) and percentage errors for probe responses.

	Attend intact	Attend split	Uncued intact	Uncued split	Unprimed intact	Unprimed split
M	699	712	793	812	824	806
SE	79	88	87	89	93	94
% error	8 +/− 1	9 +/− 1	9 +/− 1	8 +/− 1	7 +/− 1	7 +/− 1

**Table 2:** Factorial effects of Attention and Configuration on BOLD repetition effects that survived p < .05 corrected for the space defined by the object vs scrambled localiser contrast (indicated with *) or p < .10 corrected for the space defined by the intact vs split localiser contrast (indicated with ^+^).

*Contrast*	*Region*	*MNI coordinates*			*Z*
Main effect of Attention	Ant fusiform gyrus L	− 36	− 36	− 21	3.83*
Main effect of Configuration	Sup parietal R	+ 36	− 54	+ 57	3.48^+^
Interaction Attention × Configuration	Middle occipital gyrus L	− 51	− 66	− 12	3.80*

## Footnotes

1 Indeed, there was no reliable correlation between amount of priming and BOLD repetition effects for the attended-intact condition, r = −.20, p = .43.

2 Correlations between visual object RS and behavioural priming have often not been found (Sayres and Grill-Spector, 2006; Xu et al., 2007; Horner and Henson, 2008; Andresen et al., 2009; though see Ganel et al., 2006). However, these previous studies have tended to measure priming with a semantic classification task, for which a large part of the priming-related variance in RTs is likely to be explained by stimulus–response bindings, rather than by access to visual object representations (Dobbins et al., 2004; Horner and Henson, 2008).

3 This is not to say that the representations mediating view-specific effects are based on contours. Similar to Hayworth and Biederman's (2006) results, Kourtzi and Kanwisher (2000) found adaptation in the lateral occipital cortex even when contours differed between equivalently perceived shapes of a repeated object, but not when contours were identical but perceived shape differed. These data indicate that the lateral occipital cortex represents not simple image features, but rather higher level shape information, which may explain why previous researchers observed scale independence in this region (Vuilleumier et al., 2002).

4 This match might even occur for intact primes that were not cued, as long as they were registered into VSTM in some way. Uncued primes might enter VSTM via leakage of attention to the uncued location, as might be encouraged by the present priming paradigm, given that uncued primes could potentially still help the naming of the probe on one third of trials. By using catch trials however, Thoma and Davidoff (2006) showed that none of their participants were able to remember uncued stimuli in a similar priming paradigm, rendering this attentional-slippage argument unlikely.

5 Such interactions between dorsal and ventral streams might occur too rapidly to be detectable with fMRI, but might be detected with the higher temporal resolution of EEG or MEG data.

## References

[bb0005] Andresen D.R., Vinberg J., Grill-Spector K. (2009). The representation of object viewpoint in human visual cortex. Neuroimage.

[bb0010] Bartram D.J. (1976). Levels of coding in picture–picture comparison tasks. Mem. Cognit..

[bb0015] Basri R., Ullman S. (1993). The alignment of objects with smooth surfaces. CVGIP Image Underst..

[bb0020] Biederman I. (1987). Recognition-by-components: a theory of human image understanding. Psychol. Rev..

[bb0025] Biederman I. (2000). Recognizing depth-rotated objects: a review of recent research and theory. Spat. Vis..

[bb0030] Biederman I., Bar M. (1999). One-shot viewpoint invariance in matching novel objects. Vision Res..

[bb0035] Biederman I., Cooper E.E. (1991). Priming contour-deleted images: evidence for intermediate representations in visual object recognition. Cogn. Psychol..

[bb0040] Biederman I., Gerhardstein P.C. (1993). Recognizing depth-rotated objects: evidence and conditions for three-dimensional viewpoint invariance. J. Exp. Psychol. Hum. Percept. Perform..

[bb0045] Bruce V., Carson D., Burton A.M., Ellis A.W. (2000). Perceptual priming is not a necessary consequence of semantic classification of pictures. Q. J. Exp. Psychol. A.

[bb0050] Bulthoff H.H., Edelman S. (1992). Psychophysical support for a two-dimensional view interpolation theory of objectrecognition. Proc. Natl. Acad. Sci. U. S. A..

[bb0055] Burgund E.D., Marsolek C.J. (2000). Viewpoint-invariant and viewpoint-dependent object recognition in dissociable neural subsystems. Psychon. Bull. Rev..

[bb0060] Cave C.B., Kosslyn S.M. (1993). The role of parts and spatial relations in object identification. Perception.

[bb0065] Chouinard P., Morrissey B., Kohler S., Goodale M. (2008). Repetition suppression in occipital–temporal visual areas is modulated by physical rather than semantic features of objects. Neuroimage.

[bb0070] Cycowicz Y.M., Friedman D., Rothstein M., Snodgrass J.G. (1997). Picture naming by young children: norms for name agreement, familiarity, and visual complexity. J. Exp. Child Psychol..

[bb0075] Davidoff J., Warrington E.K. (1999). The bare bones of object recognition: implications from a case of object recognition impairment. Neuropsychologia.

[bb0080] Dien J. (2009). A tale of two recognition systems: implications of the fusiform face area and the visual word form area for lateralized object recognition models. Neuropsychologia.

[bb0085] Dobbins I.G., Schnyer D.M., Verfaellie M., Schacter D.L. (2004). Cortical activity reductions during repetition priming can result from rapid response learning. Nature.

[bb0090] Dolan R.J., Fink G.R., Rolls E., Booth M., Holmes A., Frackowiak R.S., Friston K.J. (1997). How the brain learns to see objects and faces in an impoverished context. Nature.

[bb0095] Edelman S., Intrator N. (2000). (Coarse coding of shape fragments) + (retinotopy) ≈ representation of structure. Spat. Vis..

[bb0100] Edelman S., Intrator N. (2003). Towards structural systematicity in distributed, statically bound visual representations. Cogn. Sci..

[bb0105] Eger E., Henson R.N., Driver J., Dolan R.J. (2004). BOLD repetition decreases in object-responsive ventral visual areas depend on spatial attention. J. Neurophysiol..

[bb0110] Eger E., Henson R., Driver J., Dolan R. (2007). Mechanisms of top–down facilitation in perception of visual objects studied by fMRI. Cereb. Cortex.

[bb0115] Epstein R.A., Parker W.E., Feiler A.M. (2008). Two kinds of fMRI repetition suppression? Evidence for dissociable neural mechanisms. J. Neurophysiol..

[bb0120] Eriksen C.W., St James J.D. (1986). Visual attention within and around the field of focal attention: a zoom lens model. Percept. Psychophys..

[bb0125] Ewbank M.P., Schluppeck D., Andrews T.J. (2005). fMR-adaptation reveals a distributed representation of inanimate objects and places in human visual cortex. Neuroimage.

[bb0130] Fang F., He S. (2005). Cortical responses to invisible objects in the human dorsal and ventral pathways. Nat. Neurosci..

[bb0135] Fang F., Murray S.O., He S. (2006). Duration-dependent fMRI adaptation and distributed viewer-centered face representation in human visual cortex. Cereb. Cortex.

[bb0145] Friston K.J., Glaser D.E., Henson R.N.A., Kiebel S., Phillips C., Ashburner J. (2002). Classical and Bayesian inference in neuroimaging: applications. Neuroimage.

[bb0150] Friston K., Rotshtein P., Geng J., Sterzer P., Henson R. (2006). A critique of functional localisers. Neuroimage.

[bb0155] Ganel T., Gonzalez C.L., Valyear K.F., Culham J.C., Goodale M.A., Köhler S. (2006). The relationship between fMRI adaptation and repetition priming. Neuroimage.

[bb0160] Gauthier I., Hayward W.G., Tarr M.J., Anderson A.W., Skudlarski P., Gore J.C. (2002). BOLD activity during mental rotation and viewpoint-dependent object recognition. Neuron.

[bb0165] Goodale M.A., Milner A.D. (2004). Plans for action. Behav. Brain Sci..

[bb0170] Grabowska A., Nowicka A. (1996). Visual spatial frequency model of cerebral asymmetry: a critical surwey of behavioural and elecrophysiological studies. Psychol. Bull..

[bb0175] Grill-Spector K., Kushnir T., Edelman S., Avidan G., Itzchak Y., Malach R. (1999). Differential processing of objects under various viewing conditions in the human lateral occipital complex. Neuron.

[bb0180] Grill-Spector K., Henson R., Martin A. (2006). Repetition and the brain: neural models of stimulus-specific effects. Trends Cogn. Sci. (Regul. Ed.).

[bb0185] Hayward W.G., Zhou G., Man W., Harris I.M. (2010). Repetition blindness for rotated objects. J. Exp. Psychol. Hum. Percept. Perform..

[bb0190] Hayworth K.J., Biederman I. (2006). Neural evidence for intermediate representations in object recognition. Vision Res..

[bb0195] Henson R. (2003). Neuroimaging studies of priming. Prog. Neurobiol..

[bb0200] Henson R., Mouchlianitis E. (2007). Effect of spatial attention on stimulus-specific haemodynamic repetition effects. Neuroimage.

[bb0205] Henson R., Rylands A., Ross E., Vuilleumeir P., Rugg M. (2004). The effect of repetition lag on electrophysiological and haemodynamic correlates of visual object priming. Neuroimage.

[bb0450] Horner, A.J., Henson, R.N., in press. Repetition suppression in occipitotemporal cortex despite negligible visual similarity: evidence for post-perceptual processing? Hum Brain Mapp. 10.1002/hbm.21124 PMC6870074 20814963

[bb0210] Horner A., Henson R. (2008). Priming, response learning and repetition suppression. Neuropsychologia.

[bb0215] Hummel J.E. (2001). Complementary solutions to the binding problem in vision: implications for shape perception and object recognition. Vis. Cogn..

[bb0455] Hummel J.E., 2003. “Effective systematicity” in, “effective systematicity” out: a reply to Edelman and Intrator (2003). Cog. Sci. 27:327–329.

[bb0220] Hummel J.E., Biederman I. (1992). Dynamic binding in a neural network for shape recognition. Psychol. Rev..

[bb0225] Hummel J.E., Stankiewicz B.J., Inui T., McClelland J. (1996). An architecture for rapid, hierarchical structural description. Attention and Performance XVI: Information Integration in Perception and Communication.

[bb0230] Humphreys G.W., Riddoch M.J., Quinlan P.T., Price C.J., Donnelly N. (1992). Parallel pattern processing and visual agnosia. Can. J. Psychol. Rev. Can. Psychol..

[bb0240] James T.W., Humphrey G., Gati J.S., Menon R.S., Goodale M.A. (2002). Differential effects of viewpoint on object-driven activation in dorsal and ventral streams. Neuron.

[bb0245] Kourtzi Z., Kanwisher N. (2000). Cortical regions involved in perceiving object shape. J. Neurosci..

[bb0250] Koutstaal W., Wagner A.D., Rotte M., Maril A., Buckner R.L., Schacter D.L. (2001). Perceptual specificity in visual object priming: functional magnetic resonance imaging evidence for a laterality difference in fusiform cortex. Neuropsychologia.

[bb0255] Lachter J., Forster K.I., Ruthruff E. (2004). Forty-five years after Broadbent (1958): still no identification without attention. Psychol. Rev..

[bb0260] Lawson R. (1999). Achieving visual object constancy across plane rotation and depth rotation. Acta Psychol..

[bb0265] Layman S., Greene E. (1988). The effect of stroke on object recognition. Brain Cogn..

[bb0270] Lazareva O.F., Wasserman E.A., Biederman I. (2008). Pigeons and humans are more sensitive to nonaccidental than to metric changes in visual objects. Behav. Processes.

[bb0275] Logothetis N.K., Pauls J., Bulthoff H.H., Poggio T. (1994). View-dependent object recognition by monkeys. Curr. Biol..

[bb0280] Lowe D. (1985). Perceptual organization and visual recognition. Distributors for North America Kluwer Academic Publishers, Boston.

[bb0285] Marr D., Nishihara H.K. (1978). Representation and recognition of the spatial organization of three-dimensional shapes. Proc. R. Soc. B Biol. Sci..

[bb0290] Marsolek C.J. (1999). Dissociable neural subsystems underlie abstract and specific object recognition. Psychol. Sci..

[bb0295] Milner A., Goodale M.A. (1995). The Visual Brain in Action.

[bb0300] Murray S.O., Wojciulik E. (2003). Attention increases neural selectivity in the human lateral occipital complex. Nat. Neurosci..

[bb0305] Newell F.N., Sheppard D.M., Edelman S., Shapiro K.L. (2005). The interaction of shape- and location-based priming in object categorisation: evidence for a hybrid “what + where” representation stage. Vision Res..

[bb0310] Nichols T., Hayasaka S. (2003). Controlling the familywise error rate in functional neuroimaging: a comparative review. Stat. Methods Med. Res..

[bb0315] Peissig J.J., Tarr M.J. (2007). Visual object recognition: do we know more now than we did 20 years ago?. Annu. Rev. Psychol..

[bb0320] Perrett D., Oram M.W., Ashbridge E. (1998). Evidence accumulation in cell populations responsive to faces: an account of generalisation of recognition without mental transformations. Cognition.

[bb0325] Poggio T., Edelman S. (1990). A network that learns to recognize three-dimensional objects. Nature.

[bb0330] Saiki J., Hummel J.E. (1996). Attribute conjunctions and the part configuration advantage in object category learning. J. Exp. Psychol. Learn. Mem. Cogn..

[bb0335] Saxe R., Brett M., Kanwisher N. (2006). Divide and conquer: a defense offunctional localizers. Neuroimage.

[bb0340] Sayres R., Grill-Spector K. (2006). Object-selective cortex exhibits performance-independent repetition suppression. J. Neurophysiol..

[bb0345] Simons J.S., Koutstaal W., Prince S., Wagner A.D., Schacter D.L. (2003). Neural mechanisms of visual object priming: evidence for perceptual and semantic distinctions in fusiform cortex. Neuroimage.

[bb0350] Snodgrass J.G., Vanderwart M. (1980). A standardized set of 260 pictures: norms for name agreement, image agreement, familiarity, and visual complexity. J. Exp. Psychol. Hum. Learn..

[bb0355] Stankiewicz B.J., Hummel J.E. (2002). Automatic priming for translation- and scale-invariant representations of object shape. Vis. Cogn..

[bb0360] Stankiewicz B.J., Hummel J.E., Cooper E.E. (1998). The role of attention in priming for left–right reflections of object images: evidence for a dual representation of object shape. J. Exp. Psychol. Hum. Percept. Perform..

[bb0365] Sutherland N.S. (1968). Outlines of a theory of visual pattern recognition in animals and man. Proc. R. Soc. B Biol. Sci..

[bb0370] Tarr M.J. (1995). Rotating objects to recognize them: a case study on the role of viewpoint dependency in the recognition of three-dimensional objects. Psychon. Bull. Rev..

[bb0375] Tarr M.J., Gauthier I. (1998). Do viewpoint-dependent mechanisms generalize across members of a class?. Cognition.

[bb0380] Thoma V., Davidoff J. (2006). Priming of depth-rotated objects depends on attention and part changes. Exp. Psychol..

[bb0385] Thoma V., Davidoff J.B., Osaka I., Rentschler I., Biederman I. (2007). Object recognition: attention and dual routes. Object Recognition, Attention & Action. Tokyo.

[bb0390] Thoma V., Hummel J.E., Davidoff J. (2004). Evidence for holistic representations of ignored images and analytic representations of attended images. J. Exp. Psychol. Hum. Percept. Perform..

[bb0395] Thoma V., Davidoff J., Hummel J. (2007). Priming of plane-rotated objects depends on attention and view familiarity. PVIS.

[bb0400] Todd J.J., Marois R. (2004). Capacity limit of visual short-term memory in human posterior parietal cortex. Nature.

[bb0405] Ullman S. (1989). Aligning pictorial descriptions: an approach to object recognition. Cognition.

[bb0410] Ullman S. (1998). Three-dimensional object recognition based on the combination of views. Cognition.

[bb0415] Ullman S. (2007). Object recognition and segmentation by a fragment-based hierarchy. Trends Cogn. Sci..

[bb0420] Vuilleumier P., Henson R.N., Driver J., Dolan R.J. (2002). Multiple levels of visual object constancy revealed by event-related fMRI of repetition priming. Nat. Neurosci..

[bb0425] Vuilleumier P., Schwartz S., Duhoux S., Dolan R.J., Driver J. (2005). Selective attention modulates neural substrates of repetition priming and “implicit” visual memory: suppressions and enhancements revealed by fMRI. J. Cogn. Neurosci..

[bb0430] Warrington E.K., James M. (1988). Visual apperceptive agnosia: a clinico-anatomical study of three cases. Cortex.

[bb0435] Warrington E.K., Taylor A.M. (1978). Two categorical stages of object recognition. Perception.

[bb0460] Xu, Y., Chun, M.M., 2006. Dissociable neural mechanisms supporting visual short-term memory for objects. Nature 440, 91–95. 10.1038/nature04262 16382240

[bb0440] Xu Y., Turk-Browne N.B., Chun M.M. (2007). Dissociating task performance from fMRI repetition attenuation in ventral visual cortex. J. Neurosci..

[bb0445] Zago L., Fenske M.J., Aminoff E., Bar M. (2005). The rise and fall of visual priming: how visual exposure shapes cortical representations of objetcs. Cereb. Cortex.

