# Supplementary material for: Object representations in ventral and dorsal visual streams: fMRI repetition effects depend on attention and part–whole configuration
Source: Neuroimage. 2011 Jul 15;57(2):513–25. doi: 10.1016/j.neuroimage.2011.04.035 (PMC3176913; doi:10.1016/j.neuroimage.2011.04.035)

Supplementary Material for:

Object Representations in Ventral and Dorsal Visual Streams: fMRI Repetition Effects Depend on Attention and Part-whole Configuration

Volker Thoma

School of Psychology, University of East London, UK

Richard N. Henson

MRC Cognition and Sciences Unit, Cambridge, UK

Corresponding author: Volker Thoma.

Email: v.thoma@uel.ac.uk

Tel.: +44 (0)20 8223 4438

Fax: +44 (0)20 8223 4937

Address: School of Psychology, UEL, Water Lane, E15 4LZ, London, UK.

Supplementary Table 1: Highest four homologous local maxima within clusters that survived p<.05 corrected for their spatial extent (using a height threshold of p<.001 uncorrected) in the contrast of objects (averaging over intact and split) versus scrambled objects in the localiser session (see also Figure 3A in paper). Only three clusters showed the opposite pattern of greater responses to scrambled objects at this threshold, two right occipital clusters (one with a maximum at [+30 -87 +24], Z = 4.94), the other with maximum at [+24 -81 -9], Z = 4.62) and one right inferior frontal cluster (with maximum at [+30 +21 -3], Z=4.44).

Region		*MNI coordinates*		*#voxels*	*Z*
Left Inferior Occipital Gyrus	-48	-81	+9	998	4.96
Left Inferior Temporal Gyrus	-51	-75	-6		4.94
Left Mid Fusiform Gyrus	-42	-48	-18		4.89
Left Parahippocampal Gyrus	-36	-27	-21		4.66
Right Inferior Occipital Gyrus	+54	-66	+12	856	5.27
Right Inferior Temporal Gyrus	+48	-72	-6		5.79
Right Mid Fusiform Gyrus	+45	-51	-18		4.95
Right Parahippocampal Gyrus	+33	-21	-18		4.97

Supplementary Table 2: Highest three homologous maxima within clusters that survived $p<.05$ corrected for their spatial extent (using a height threshold of $p<.001$ uncorrected) in the contrast of split versus intact objects in the localiser session (see also Figure 3B in paper). No cluster showed greater responses to intact than split objects at this threshold.

Region		*MNI coordinates*		*#voxels*	*Z*
Left Superior Occipital Gyrus	-33	-81	+18	881	5.46
Left Inferior Temporal Gyrus	-39	-69	-6		3.95
Left Intraparietal Sulcus	-24	-57	+51		4.90
Right Superior Occipital Gyrus	+33	-78	+21	1296	5.95
Right Inferior Temporal Gyrus	+45	-60	-9		5.19
Right Intraparietal Sulcus	+27	-48	+54		4.70

Supplementary Table 3: Additional voxels that survived the same SVC correction-level as in the main paper, but outside the localiser masks.

Effect/Region	*MNI coordinates*	*#voxels*	*Z*
Main Effect of Attention			
Left precentral gyrus	-54 -6 +42	13	4.65
Main Effect of Attention			
Left precentral gyrus	-54 -6 +42	13	4.65
Attention X Configuration			
Left medial prefrontal	-6 +24 +45	3	3.81

Supplementary Table 4: Results of additional tests of the four simple effects of repetition (priming) within each condition (that survived the same SVC correction as for either of the localiser contrasts as in the main paper: * = p<.05, two-tailed, corrected for the space defined by the object vs scrambled localiser contrast; + = p<.05, two-tailed, corrected for the space defined by the intact vs split localiser contrast).

Effect/Region	*MNI coordinates*	*#voxels*	*Z*
Cued Intact			
Left Mid Fusiform Gyrus	-42 -48 -12	129	5.01*
Left Inferior Temporal Gyrus	-45 -69 -12		5.00*/+
Right Intraparietal Sulcus	+39 -51 +51	38	4.52+
Left superior occipital gyrus	-18 -93 +30	9	4.49+
Cued Split			
-	-	-	-
Uncued Intact			
-	-	-	-
Uncued Split			
-	-	-	-

Supplementary Figure 1: Voxel-wise repetition effects but now relative to voxels showing a reliable difference (corrected for extent given a height threshold of $p<.001$ uncorrected, as for the localiser contrasts) between attention to left (green voxels) and right (purple voxels) primes during the main experiment. The top left panel shows a MIP of the F-contrast comparing left and right primes (see Figure 3 legend for more details). The remaining panels show sections through the peak of the red voxels that showed a main effect of attention (bottom left), main effect of configuration (bottom right) and interaction between attention and configuration (top right) (see Figure 4 legend for more details).

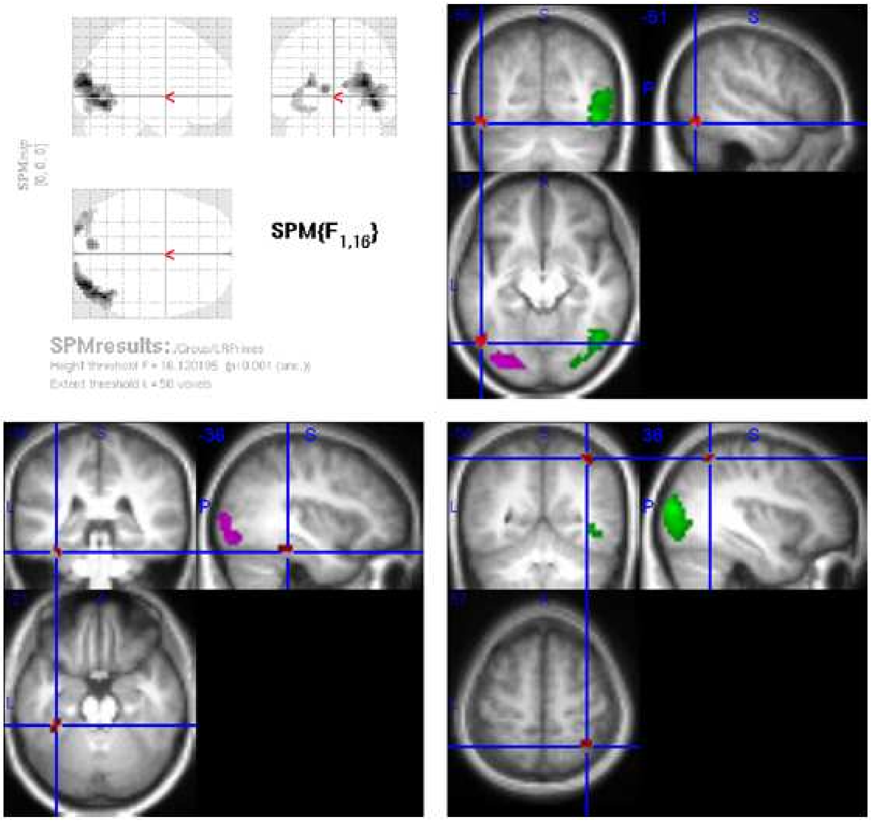

Individually-defined fROIs

Following a request from a reviewer, the data were also analysed by defining fROIs on an individual basis, rather than on a group basis (as in the main paper). Defining fROIs individually is a common practice for regions like the Lateral Occipital Complex (LOC) (Grill-Spector et al., 1999), and allows for possible anatomical variability across participants in the functional organisation of their brains (see, e.g., Saxe, Brett, & Kanwisher, 2006, for further discussion; though see also conclusion below).

To identify individual fROIs, the SPMs for the object vs scrambled and split versus intact T-contrasts within each participant's (first-level) GLM for the localiser session were thresholded at $p<.05$ uncorrected, and the maximum that was closest to the maximum of the corresponding fROI in the group (second-level) analysis (as shown in Supplementary Tables 1-2) was selected (note that the individual data were still anatomically-normalised to a template brain, as in Methods). In order to restrict the individual maxima to approximately the same anatomical region, the largest Euclidean between the individual and group maxima was set at 34mm, which was sufficient to ensure that all individual maxima were within the appropriate hemisphere at least. This meant the exclusion of two participants for the dorsal stream analysis, who did not have a maximum within 34mm of the left intraparietal group maximum (and for whom a limit greater than 34mm resulted in maxima in the right hemisphere instead). The mean, minimum and maximum of these individual MNI coordinates are shown in Supplementary Table 3. Having identified maxima for individual participants, the data were extracted by averaging data from voxels within 8mm of each such maxima (as done for data extraction for the group fROIs in the main paper).

Individual-fROI approach – ventral visual stream

For the four ventral visual fROIs defined by the object vs scrambled localiser contrast, the 2x2x2x2 ANOVA on BOLD repetition effects revealed, as in the group-fROI analysis in the main paper, a reliable interaction between Attention and Laterality, $F(1,16)=10.2$, $p<.01$ (in addition the expected main effect of Attention, $F(1,16)=4.60$, $p<.05$, though the main effect of laterality no longer reached significance, $F(1,16)=2.82$, $p=.11$). The corresponding plot of the mean repetition effects corresponding to this interaction (i.e, averaging across the factors of Configuration and Rostrality) showed reliable RS in Attended conditions in the left hemisphere, but not the right (Supplementary Figure 2, left panel), as in Figure 5A. This was confirmed by separate ANOVAs on left and right hemisphere fROIs, which showed a reliable main effect of Attention in the left, $F(1,16)=13.1$, $p<.005$, but not right, $F(1,16)<1$, hemisphere. The three-way interaction between Rostrality, Laterality and Configuration that reached significance in the group-fROI analysis did not quite reach significance for the individually-defined fROIs, $F(1,16)=3.91$, $p=.066$.

As with the group-fROI analysis, there was a reliable positive correlation across participants between their amount of priming and their amount RS for Attended objects in the left hemisphere fROIs (averaged across Rostrality and Configuration), Pearson's $R=0.64$, $p<.01$ (Supplementary Figure 2, right panel), as in Figure 5A. There was no such correlation for right hemisphere fROIs, or for Uncued conditions, $Rs<.25$, $ps>.33$.

Individual-fROI approach – dorsal visual stream

A left intraparietal maximum could not be found at $p<.05$ uncorrected for the split vs intact localiser contrast in two of the seventeen participants (see above). In the 2x2x2 ANOVA with factors Laterality, Attention and Configuration for group-fROIs that is reported in the main paper, there was a reliable main effect of Configuration and Laterality. The main effect of Configuration for the present individual-fROIs

approached significance, F(1,14)=3.85, p=.070, but the same was not true of the main effect of Laterality, F(1,14)=2.38, p=.14. Unlike with the group-fROIs, however, the interaction between Attention and Configuration also approached significance, F(1,14)=4.17, p=.060. This interaction appeared to reflect a greater difference between Intact and Split repetition effects for Uncued than Attended objects (Supplementary Figure 3; left panel); indeed, the only repetition effect that was reliable when averaging across Hemisphere was RE in the Intact Uncued condition. Like with the group-fROIs though, the only repetition effect that was reliable when averaging across Attention was in the right fROI (Supplementary Figure 3, right panel; consistent with Figure 5B).

Finally, as with the group-fROIs, there was a reliable positive correlation across participants between their behavioural priming and their amount of RE for Uncued Intact primes, R=.539, p<.05 (see Supplementary Figure 4), but not for Uncued, Split primes, R=-0.26, p=.34.

Summary of individual fROI results

In general, these results from individually-defined fROIs are similar to those reported in the main paper for group-defined fROIs. Those differences are mainly in some ANOVA effects not reaching the same level of significance as for the group-fROI ANOVAs (and in the case of the dorsal stream fROIs, this may reflect reduced statistical power, given that bilateral intraparietal maxima could not be identified for two of the participants, though an additional interaction between Attention and Configuration was also revealed here). Importantly, both of the correlations between BOLD repetition effects and behavioural priming in the ventral and dorsal stream regions are still reliable.

The more general question of whether group-defined or individually-defined fROIs are more sensitive depends not only on the consistency of the functional-anatomical mapping across individuals, but also on the relative size of within-participant (between-scan) variability versus between-participant variability (see also Henson & Mouchlianitis, 2007, Supplementary Material). Given the similarity of the results using the two approaches here, it seems likely that the present functional effects occurred in similar anatomical regions for all participants (following anatomical normalisation to a template brain, and smoothing by 10mm to allow for residual anatomical differences beyond the ability of normalisation to match). In this case, effects that are more significant in the group-defined than individually-defined fROIs may reflect relatively large within-participant variability (relative to between-participant variability). It must be remembered that this within-participant variability includes error in the estimation of coordinates of the maxima from the localiser scans within individual participants (i.e, the coordinates of their true fROI could be many millimetres removed, even relative to the 8mm-spherical averaging, owing to noise in the estimation of the maximal T-value). This precision of the estimated fROI coordinates depends on the signal-to-noise ratio (SNR) in the localiser data, which in turn depends on factors like the between-scan (within-participant) variability and the number of scans. If this precision is low, i.e, localisation error is large (and the functional-anatomical mapping is consistent across participants), then a more accurate estimate of each individual's fROI coordinates can be obtained from the group-maximum coordinates (by virtue of pooling across more data). While this may be the case in the present data, it should also be remembered that, if the precision of fROI estimation in individual participants is high (i.e, high SNR localiser data), and the consistency of the functional-anatomical mapping across participants is low (e.g, for functions different from those considered here), then individually-defined fROI will be more sensitive than group-defined fROI.

Supplementary Table 3: Mean, minimal and maximum of MNI coordinates across participants for six functional regions of interest (fROI) when identified from maxima that survived p<.05 uncorrected in the localiser contrast of objects versus scrambled (four ventral stream fROIs) or split versus intact (two dorsal stream fROIs) in individual participant analyses. Maxima were constrained to lie within 34mm of the group-defined maximum in Supplementary Tables 1 and 2 (to ensure within approximately the same anatomical region; particularly the same hemisphere). This meant the exclusion of two participants in whom no significant maximum was found for the left dorsal fROI.

fROI		*MNI coordinates*	
		Mean/min/max	
Left posterior ventral	-47	-74	-2
(Left Inferior Temporal Gyrus)	-54	-81	-12
	-42	-47	+6
Right posterior ventral	+49	-71	-4
(Right Inferior Temporal Gyrus)	+57	-78	-15
	+57	-66	+3
Left anterior ventral	-40	-47	-17
(Left Mid-Fusiform Gyrus)	-45	-54	-24
	-36	-42	-9
Right anterior ventral	+43	-51	-18
(Right Mid-Fusiform Gyrus)	+36	-72	-33
	+54	-36	0
Left dorsal (n=15)	-26	-60	+53
(Left intraparietal)	-39	-81	+39
	-18	-48	+69
Right dorsal (n=15)	+31	-50	+53
(Right intraparietal)	+21	-63	+45
	+42	-42	+66

Supplementary Figure 2: On the left, BOLD repetition effects for Attended and Uncued conditions (averaged across Intact and Split configurations) for left (L) and right (R) hemispheric fROIs within the ventral visual stream (averaged across Posterior and Anterior fROIs; see Supplementary text). Error bars are two-tailed 95% confidence intervals of repetition effects versus zero (see Figure 5 legend for further details).
On the right, scatter plot of each participant's behavioural priming for Attended primes (averaged across Intact and Split configurations) against RS in left fROIs (averaged across posterior and anterior regions).

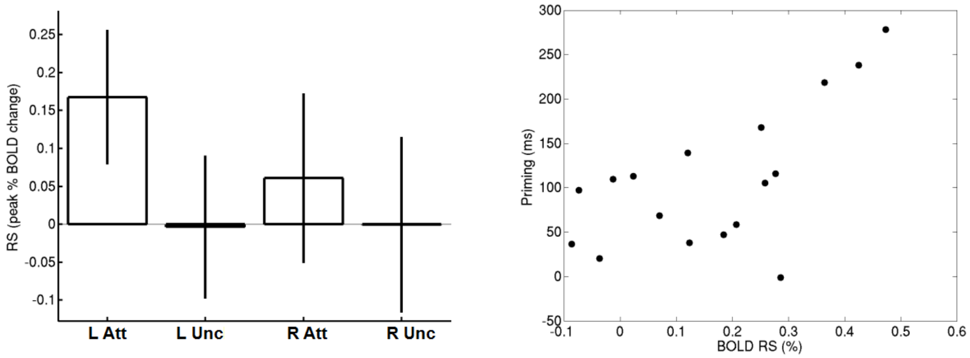

Supplementary Figure 3: BOLD repetition suppression (RS) effects for left (L) and right (R) hemispheric intraparietal individually-defined fROIs within the dorsal visual stream. The panel on left shows repetition effects for Intact and Split configurations as a function of Attended versus Uncued (averaging across Hemisphere), given the trend for an interaction between Configuration and Attention (see Supplementary text). The panel on the left shows instead repetition effects for Intact and Split configurations as a function of Hemisphere (averaging across Attention), to match the data shown in Figure 5B. See Figure 5 legend for more details.

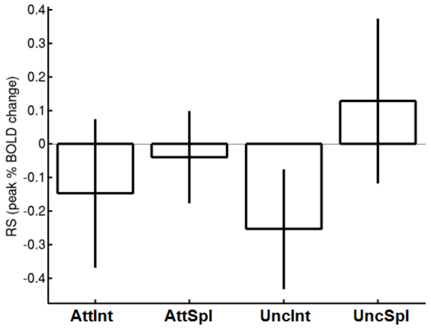

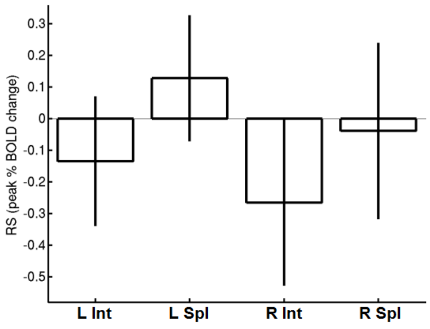

Supplementary Figure 4: Scatter plot of each participant's behavioural priming against BOLD repetition enhancement (RE) for Uncued, Intact primes in dorsal fROIs (averaged across left and right intraparietal regions). See Figure 5B legend for more details.

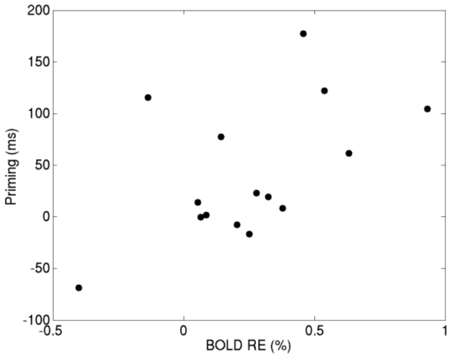

Supplementary Figure 5: Scatter plot of each participant's behavioural priming against BOLD repetition enhancement (RE) for Attended-Intact primes in dorsal fROIs.

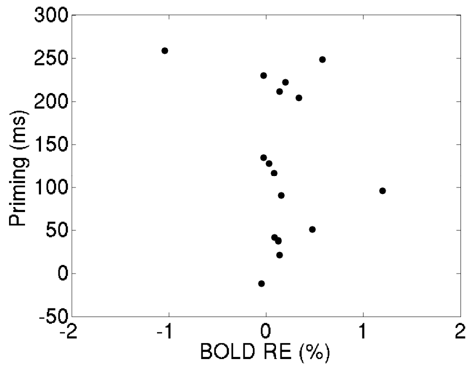

Supplement: Supplementary file 1 — Supplementary Materials. [file mmc1.pdf]
